# An Immunosensor for the Determination of Cortisol in Serum and Saliva by Array SPRi

**DOI:** 10.3390/s22249675

**Published:** 2022-12-10

**Authors:** Anna Sankiewicz, Lukasz Oldak, Beata Zelazowska-Rutkowska, Adam Hermanowicz, Zenon Lukaszewski, Ewa Gorodkiewicz

**Affiliations:** 1 Bioanalysis Laboratory, Faculty of Chemistry, University of Bialystok, Ciolkowskiego 1K, 15-245 Bialystok, Poland; 2 Doctoral School of Exact and Natural Science, Faculty of Chemistry, University of Bialystok, Ciolkowskiego 1K, 15-245 Bialystok, Poland; 3 Department of Pediatric Laboratory Diagnostics, Medical University of Bialystok, Waszyngtona 17, 15-274 Bialystok, Poland; 4 Department of Pediatric Surgery and Urology, Medical University of Bialystok, Waszyngtona 17, 15-274 Bialystok, Poland; 5 Faculty of Chemical Technology, Poznan University of Technology, 5 M. Skłodowska-Curie Square, 60-965 Poznan, Poland

**Keywords:** SPRi array, QCM, serum, saliva, cortisol

## Abstract

Cortisol is a hormone which plays an essential role in the immune, endocrine, cardiovascular, renal and skeletal systems. Its level increases in response to stress, illness, injury or exhaustion, and it is therefore a significant diagnostic biomarker of stress. An immunosensor for the determination of cortisol by SPRi array was developed. The receptive part of the immunosensor is mouse monoclonal antibody against cortisol, immobilized via cysteamine linker. The optimum pH of the immunosensor is 7.4, and the optimum concentration of the antibody is 50 ng mL^−1^. The immunosensor is specific for cortisol, and its linear response ranges from 0.20 ng mL^−1^ (LOQ) to 8 ng mL^−1^. The precision of the determination was between 3.1% and 3.3%, and the recovery between 99% and 102%. The immunosensor was validated by simultaneous determination of cortisol in serum and saliva samples by a standard method, with good agreement between the results.

## 1. Introduction

Cortisol is a hormone which plays an essential role in the immune, endocrine, cardiovascular, renal and skeletal systems. Its level increases in response to stress, illness, injury or exhaustion, and it is therefore a significant diagnostic biomarker of stress. The hormone belongs to the group of glucocorticosteroids. It is a steroid hormone produced in the zona fasciculata of the adrenal cortex. Its secretion is subject to the circadian cycle. Its concentration in body fluids is highest in the morning and lowest around midnight [1,2]. The normal cortisol level in serum varies from 30 to 140 ng mL^−1^ [3]. Stress, night work and illness can disrupt the circadian rhythm of cortisol. In the blood, 90–95% is bound to proteins, with 80% is bound to globulins and 10–15% to albumin, while 5–10% of cortisol is in the free form. The bound form is biologically inactive and creates a reserve [4].

Cortisol concentration can be measured in blood serum or plasma, urine, saliva, cerebrospinal fluid and sweat [5,6,7]. Cortisol levels measured in plasma or serum lead to an estimate of the total concentration. The result of analysis of the cortisol level in other biological materials will be the concentration of free, unbound cortisol only. In particular, the measurement of cortisol in saliva appears to be important. Salivary cortisol is an ultrafiltrate of plasma cortisol and is not bound to protein as it is in blood. A strongly positive correlation between salivary and blood cortisol levels has been reported [8].

For the determination of cortisol, diagnostic laboratories normally use different commercial kits and automated platforms based on immunoassay (IA) tests. The most frequently used are enzyme immunoassays (EIA), enzyme-linked immunosorbent assays (ELISA), and luminescence or fluorescence tests [9]. In view of the need to use radioactive material, radioimmunoassays are less used in the routine ambulatory diagnostic procedure for evaluating cortisol levels [10]. Chromatographic methods, such as high performance liquid chromatography (HPLC) with UV [11] or fluorescence detection [12] and tandem liquid chromatography mass spectrometry (LC-MS/MS) may be used [12,13]. The literature also describes the detection of cortisol by immunochromatographic analysis. Researchers created immunochromatographic strips for the determination of cortisol in saliva [14]. An automated electrochemiluminescence test (ECLIA) called the Roche Cortisol II test is also used [15]. There have been many reports of the use of biosensors for the detection of cortisol [16,17,18,19,20,21,22,23,24,25,26,27,28]. These are summarized in Table 1.

Electrochemical and optical biosensors are particularly widely described in the literature. A number of electrochemical techniques have been used in cortisol determination: electrochemical impedance spectroscopy [20], cyclic voltammetry [21] and miniaturized differential pulse voltammetry [23]. In all these studies, covalently immobilized anti-cortisol antibody was used as the capturing element. Various strategies were used for enhancement of the analytical signal: bovine serum albumin [20], conductive carbon yarn with elipsoidal Fe_2_O_3_ [21], and a sandwich of gold nanoparticles/molybdenum disulfide/gold nanoparticles [23].

Among the optical sensors, the method of surface plasmon resonance (SPR) is the most commonly used. Most of these biosensors are fluidic or microfluidic systems with a suitable antibody as a sensing element. The most sensitive types have, in addition to the primary antibody, a secondary antibody serving to enhance the analytical signal [16]. Several localized surface plasmon resonance (LSPR) biosensors have also been developed. LSPR sensors use gold nanoparticles immobilized on a silica round cover slip [17], polycarbonate strip [18] or plastic optical fiber [19], and in most cases are used exclusively for cortisol detection. There are several similarities between electrochemical and SPR biosensors; they include the use of anti-cortisol antibody as a sensing element, and in the various approaches used for enhancement of the analytical signal.

The SPRi Array differs from the above mentioned techniques using SPR or LSPR biosensors. This technique is designed for the quantitative determination of biomarkers in body fluids. In contrast to fluidic versions of SPR, the measurement of the SPRi signal occurs after the removal of the solution, and a biosensor is prepared ex situ—whereas in fluidic SPR the biosensor is formed in situ during measurement. A biosensor in the SPRi array consists of a large number (most typically 108) of measuring points separated by a polymeric mask. The measuring points form several measuring cells (typically nine), which enables the measurement of several samples (typically nine) simultaneously. Due to these differences from fluidic SPR, the SPRi array is able to measure concentrations of biomarkers at their normal level in body fluid without any signal enhancement or preconcentration. More than 20 SPRi array biosensors have been developed, including for such significant cancer biomarkers as HE4 [29], CEA [30] and aromatase [31]. The applicability of the developed SPRi array biosensors has been confirmed in a large number of studies, in which they were used in clinical investigations involving markers such as podoplanin in the serum and urine of transitional bladder cancer patients [32]; aromatase in the plasma of patients with bladder cancer [33]; and MMP2, UCHl-1 and 20S proteasome in the serum of patients with thermal injuries [34].

The aim of this paper is to add a biosensor for cortisol determination to the range of tools used with the SPRi array technique. The initial experiments show that mouse monoclonal antibody against cortisol, immobilized via a cysteamine linker, may be a promising solution and should be the subject of further work. Cysteamine is frequently used as a linker for antibody immobilization [29,30,31]. Formation of subsequent layers of biosensor can be observed by Quartz Cristal Microbalance measurements [35]. The developed biosensor should be characterized by typical analytical characteristics: specificity, linearity range, precision, LOD, LOQ, recovery spike and its applicability for cortisol determination in real samples should be demonstrated.

## 2. Materials and Methods

### 2.1. Materials and Reagents

Cortisol solution (Merck, Darmstadt, Germany), mouse monoclonal antibody against cortisol, leptin (ABCAM, Cambridge, UK), cysteamine hydrochloride, N-ethyl- N′-(3-dimethylaminopropyl) carbodiimide (EDC, SIGMA-ALDRICH, Steinheim, Germany), progesterone, estradiol, testosterone, cholesterol, human albumin (all SIGMA-ALDRICH, Steinheim, Germany), and N-hydroxysuccinimide (NHS) (ALDRICH, Munich, Germany) were used. The bases of the immunosensors were chips covered with a layer of gold (Ssens, Enschede, The Netherlands).

A complete list of materials and reagents is given in Appendix A.

### 2.2. Biological Material

The Ortho-Dent and Dentablue clinics in Bialystok provided saliva samples from patients with endodontic treatment planned, and the Maria Sklodowska-Curie Oncology Center provided blood samples from patients with ovarian cancer. All samples were provided after obtaining the consent of the Bioethical Committee.

### 2.3. Procedures

#### 2.3.1. Antibody Immobilization

Glass slides coated with gold were rinsed with ethanol and milli-Q water (Burlington, MA, USA) and dried under a stream of argon. Next, each slide was immersed in a 20 mM alcohol cysteamine solution for at least 12 h at room temperature. After this time, the chips with the linker monolayer were washed with absolute ethyl alcohol and milli-Q water and finally dried under an argon atmosphere. These chips were stable over several months. In the second step, an antibody activation process was performed. For this purpose, a mixture of NHS and EDC in a ratio of 1:1 in a solution of carbonate buffer at pH 8.5 was added to the antibody solution, and was thoroughly mixed. The activated antibody was applied to the active areas of a slide with immobilized cysteamine, and this was incubated for 1 h at 37 °C. After incubation, the chip was washed with water. To eliminate nonspecific adsorption, a bovine serum albumin (BSA) solution with a concentration of 1 ng mL^−1^ was used.

#### 2.3.2. SPRi Measurements

**Figure A1:**
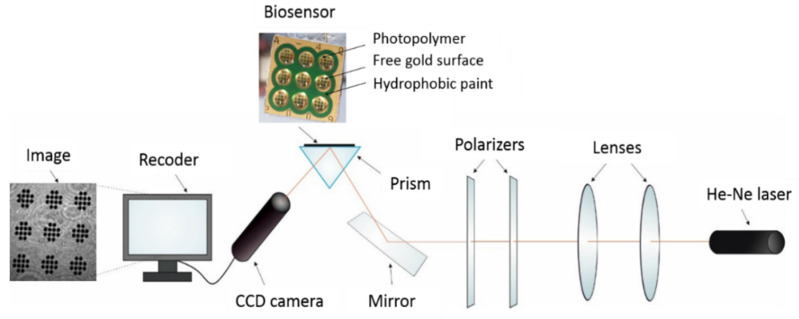
Schematic diagram of the SPRi apparatus and chip architecture.

SPRi measurements were performed using the home-made device presented in Appendix B. The apparatus and measurement chip are shown schematically in Figure A1. The measurement procedure has been described in previous papers [29,30,31]. The SPR signal was measured at a fixed SPR angle on the basis of recorded images. The angle was selected after immobilization of the receptor. Two photographs were taken (the first after antibody immobilization, the second after interaction with the cortisol). The time of antibody–cortisol interaction was 10 min. Antibody solution in PBS buffer was used, and a BSA solution was applied to block the remaining reactive free areas on the chip. To remove unbound biomolecules, the immunosensor surface was washed with HBS-ES buffer and distilled water. Each measurement was performed after thorough drying of the immunosensor surface. The SPRi signal consisted of variations of the refractive index (RI) due to biointeraction events occurring at the sensing surface, these being detected as changes in the reflected light intensity that were recorded on CCD camera image. Evaluation of the SPR images in 2D form and conversion of the numerical signals to a quantitative signal were performed using NIH Image J version 1.42 software. The SPRi signal was obtained as the difference between the signal SPRi before and after interaction with the biomolecule. A background correction was applied.

#### 2.3.3. QCM Measurements

A Quartz Crystal Microbalance (QCM, MethromAutolab B.V., Utrecht, The Netherlands) was used to confirm the formation of successive immunosensor layers. The QCM was connected to a potentiostat/galvanostat (MethromAutolab B.V. PGSTAT 302N). A crystal with a frequency of 6 MHz and a gold layer thickness of 100 nm and an area 0.361 cm^2^ was used, and placed in a measuring vessel with a maximum capacity of 3 mL. The QCM is supported by dedicated software—NOVA 2.1.

A baseline of frequency variation was determined for pure gold. Next, the quartz crystal covered with a gold layer was covered with a cysteamine monolayer by introducing an alcoholic cysteamine solution with a concentration of C = 20 mM into the measuring cell. The antibody was than activated (C = 50 ng mL^−1^) by the same procedure as that used for activation in the SPRi experiments. In the last stage, a cortisol solution with concentrations of C = 2.0; 5.0; 10.0; 15.0 and 20.0 ng mL^−1^ was introduced into the measuring cell. For each stage, changes in the resonant frequency were recorded until an equilibrium line was established.

## 3. Results and Discussion

### 3.1. Optimization of Conditions for Measurements

Mouse monoclonal antibody against cortisol was used as the receptor of the immunosensor. The optimization of the antibody concentration is described in Appendix C. The selected value of antibody concentration for further experiments was 50 ng mL^−1^.

### 3.2. Influence of Solution pH on Interaction Process

**Figure 1:**
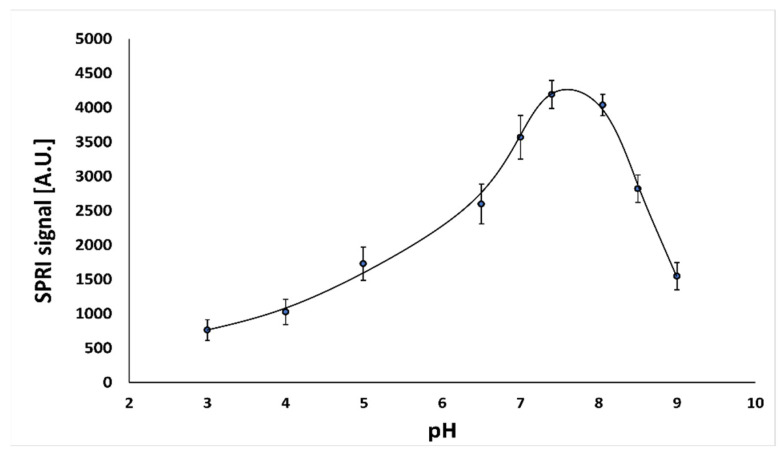
Dependence of SPRI signal (arbitrary units) for the antibody–cortisol complex on pH. The antibody concentration is 50.0 ng mL^−1^, and the cortisol concentration is 10.0 ng mL^−1^.

To test the effect of solution pH on the behavior of the immunosensor, a series of measurements for nine different pH values in the range of 3.0–9.0 was carried out. The experiments were performed at the optimal concentration of antibody (50.0 ng mL^−1^) and a constant cortisol concentration (10.0 ng mL^−1^). The results are shown in Figure 1.

The highest immunosensor response was observed at pH 7.4. To provide an optimum environment for the immunosensor, the value pH 7.4 was selected for use in all further experiments.

### 3.3. Analytical Characteristics of the Immunosensor

The analytical parameters of the immunosensor were investigated, including calibration curve, linearity range, precision, recovery, and limits of detection and quantification.

**Figure 2:**
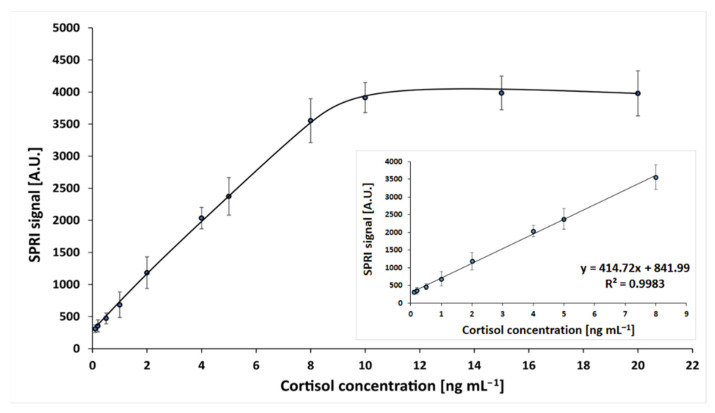
Dependence of the SPRi signal (arbitrary units) on cortisol concentration. Linear ranges are shown in an inset. The antibody concentration is 50 ng mL^−1^, and the pH of the cortisol solution is 7.4.

The dependence of the SPRi signal on cortisol concentration was investigated. Measurements were performed in the cortisol concentration range 0.1–20 ng mL^−1^, at an antibody concentration of 50 ng mL^−1^ and pH 7.4, according to the procedures described in the previous sections. The obtained calibration curve is shown in Figure 2. Standard solution samples were prepared in PBS buffer.

A curve of Langmuirian shape was obtained, and the saturation of the active points of the immunosensor was above a concentration value 10 ng mL^−1^. The linear range included concentrations from 0.1 to 8 ng mL^−1^, with regression R 2 = 0.99. The limit of detection, calculated by the formula 3.3SD/A (where A was the slope of the calibration curve), was 0.15 ng mL^−1^; and the limit of quantification (10 SD/A) was 0.20 ng mL^−1^. Thus, the assay has a useful determination range between 0.20 and 8 ng mL^−1^. The precision and recoveries of the developed immunosensor were investigated. For this purpose, four series of experiments were performed, each consisting of 24 single measurements. Cortisol concentration was measured in samples with three different spikes (0.2, 1.0 and 4.0 ng mL^−1^). These concentrations are located at the beginning, middle, and end of the of the calibration graph. The results are shown in Table 2.

According to [36], a method is accurate if the average recovery value is between 80.00% and 120.00%, and precise if the relative standard deviation (RSD) is below 20%. In our experiments the recovery was within the range 99–102%, and precision was 3.1–3.3%. Thus, the developed immunosensor achieves satisfactory precision and recovery. Worse precision and recovery (7.5% and 107%, respectively) were observed at the lowest investigated spike of cortisol, i.e., at the boundary of successful quantification.

### 3.4. Selectivity of the Immunosensor

**Figure 3:**
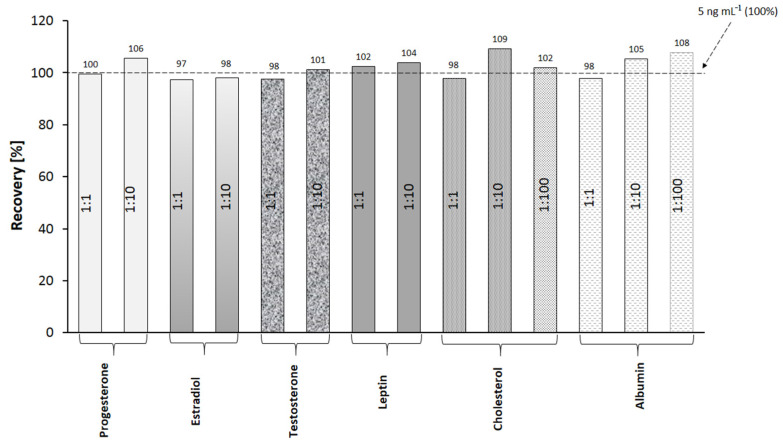
Specificity of cortisol determination. Determinations of cortisol (5 ng mL^−1^) in the presence of variable excesses of potential interferents.

Selectivity is a crucial property of methods for the determination of analytes in complex biological samples. The selectivity of the present method was evaluated by performing measurements of cortisol concentration in the presence of interferents. A series of solutions was prepared, each containing cortisol and a potential interferent in a strictly defined mass ratio. The concentration of cortisol in the prepared mixtures was 5 ng mL^−1^. The selected interferents and their mass ratios are presented in Figure 3. The methodology of immobilization and measurement is consistent with the method described in Section 2.3. The cortisol mixtures with interferents were applied to the active sites, and the SPRI signals were measured. From the results, the percentage of recovery was calculated (Figure 3).

The recovery values for the method are in the range of 97–109%, which proves the selectivity of the immunosensor in the presence of the selected interferents.

### 3.5. Testing Successive Layers of the Immunosensor

**Figure 4:**
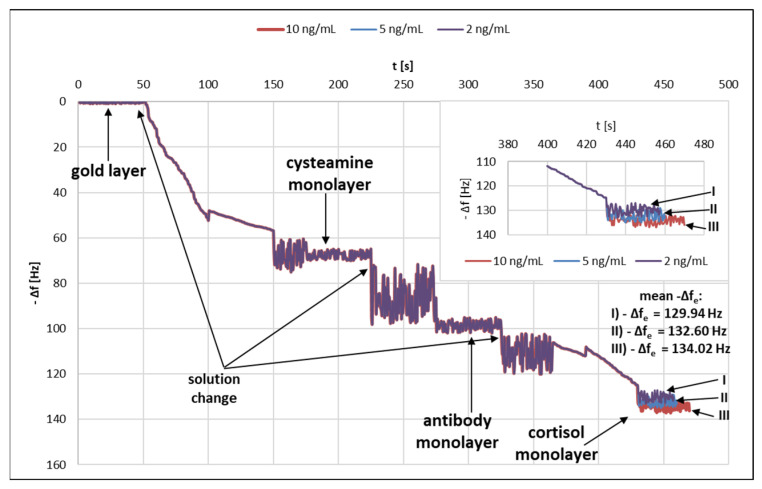
Variation of frequency as a function of time.

A quartz crystal microbalance (QCM) was used to test successive layers of the immunosensor. Figure 4 shows the graph of changes in the frequency Δf [Hz] as a function of time t [s]. Frequency changes caused by the formation of successive layers of the immunosensor were observed.

### 3.6. Application of the Immunosensor for Determination of Cortisol in Biological Samples

To verify the suitability of the developed immunosensor for cortisol determination in biological samples, several human saliva and blood serum samples were analyzed. The saliva was obtained from patients requiring endodontic treatment due to simple caries. Non-stimulated saliva was harvested just before the intervention. Twofold diluted samples were used for analysis. The blood serum samples were from ovarian cancer patients before tumor resection. The tested serum samples were diluted so that the range of signals received from the detector lay within the range of the calibration curve. The determinations were made with the developed immunosensor and by means of using a commercial analyzer (Elecsys Cortisol II, Cobas E 411, Roche, Basel, Switzerland). The results were presented in Table 3.

The cortisol concentration in selected samples obtained by SPRi and the standard method were similar. The correlation coefficient was 0.996 for serum and 0.926 for saliva. Normal levels of cortisol range 30 to 140 ng mL^−1^ in serum, and morning salivary cortisol levels range from 1 to 8 ng mL^−1^ [3] and vary slightly by diagnostic test.

## 4. Discussion

Immunoassay is the most routine and common method for the determination of cortisol in laboratories. As compared to the standard electrochemiluminescence method (Elecsys Cortisol II, Cobas E 411, Roche), the developed biosensor is a direct, label-free sensor having very simple construction, while the standard method uses a label—ruthenium complex with 2,2′-bipyridine—which, jointly with tripropylamine, is the source of the analytical signal. The determination of cortisol with the developed biosensor by the array surface plasmon resonance imaging technique joins over 25 other biosensors using this technique, and may compete with current standard methods.

Biosensors for the determination of cortisol are gaining increasing popularity. Table 1 presents examples of biosensors for the determination of cortisol in various body fluids, mostly saliva. Almost all of them enable the determination of cortisol concentrations of the order of single ng mL^−1^, characteristic for this body fluid. Observed differences in LOD for particular biosensors are insignificant, because cortisol concentrations in saliva below 1 ng mL^−1^ are not reported. An advantage of the developed biosensor is its linear calibration curve, as many of the reported biosensors listed in Table 1 use semi-logarithmic calibration curves, e.g., [26]. The precise determination of concentration on the basis of a semi-logarithmic plot is more complex than in the case of linear dependence.

The measurement of cortisol concentration using any biofluid can assist in the diagnosis and management of a variety of diseases. The measurement of cortisol in saliva can be particularly recommended as a first-line screening test, because it reflects the circadian rhythm of circulating cortisol in the body. Advantages of measuring cortisol levels in saliva include the ease of collection of the material for testing, the small volume of saliva required, and the avoidance of errors caused by stress during the collection of blood for testing. Saliva samples can be collected by patients themselves, and kept at home or sent by post directly to the laboratory. This is a significant convenience in conducting tests, especially for patients requiring frequent analyses. Besides use in humans, cortisol determination can be useful in the examination of animals. The cortisol level has effects on growth rate and feed assimilation, on traits related to robustness and adaptation, and on the animal’s resistance to bacteria and parasites [37,38]. However, blood collection is very stressful for both the animal and the sampling staff, and may therefore introduce errors that confound the assessment of the real cortisol level in animals. In short, the SPRi immunosensor presented in this paper can be used to determine cortisol concentration in human serum and saliva. It appears that the biosensor may also be useful in the examination of animals.

## 5. Conclusions

We have presented here a SPRi array immunosensor for the direct measurement of cortisol levels in serum and saliva. The immunosensor works on the basis of a highly selective interaction between immobilized mouse monoclonal antibody against cortisol and cortisol in solution, and exhibits linearity of the analytical signal response. The precision and recovery of the developed method are at acceptable levels. The results obtained with the SPRi array immunosensor for the measurement of cortisol in serum and saliva samples were consistent with a standard laboratory test. However, more samples need to be tested for the method to be fully validated.

In summary, measurements using a sensitive SPRi immunosensor are possible in samples with a highly complex matrix such as human serum and saliva, and can be performed without the need to use complicated procedures for sample preparation or to use labels as is currently required. The presented SPRi immunosensor combines the advantages of real-time response, high sensitivity, and 2D multiplex sensing. Thus the sensor show promise for applications in stress biomarker screening.

## Figures and Tables

**Table 1:** Biosensors used for cortisol determination.

Biosensor	Medium	LOD	References
Electrochemical-digital immunosensor	Saliva	0.87 ± 0.12 pg mL^−1^	[20]
Electrochemical graphene-based sensor	Sweat	0.005 fg mL^−1^	[21]
Screen-printed cortisol sensor using cyclic voltammetry as method detection	Saliva	no data	[22]
Immunosensor based on the smartphone-controlled Differential Pulse Voltammetry (DPV) system	Saliva	0.11 nM	[23]
Aptamer TISS (Target-Induced Structural Switching) nanosensor based on the aptamer	Saliva	1 nmol L^−1^	[24]
Twin sensor chip on quartz crystal micro balance (QCM)	Not tested	11 pg mL^−1^	[25]
SPR biosensor	Saliva	1.0 ng mL^−1^	[3]
Microfluidic SPR biosensor immunoassay	Saliva	49 pg mL^−1^	[16]
SPR immunosensor	Saliva, urine	3 µg L^−1^ 4 µg L^−1^	[26]
SPR immunosensor	Saliva	38 pg L^−1^	[27]
SPR biosensor	Not tested	1 ng mL^−1^	[28]
Disposable LSPR competitive biosensor	Serum	40.3 ng mL^−1^	[18]
LSPR aptasensor	Saliva	0.1 nM;	[17]
LSPR immunosensor	Not tested	1 pg mL^−1^	[19]
SPRi Array	Saliva, serum	150 pg mL^−1^	This work

**Table 2:** Precision and recovery of cortisol determination with the developed immunosensor (*n* = 24).

Series	Spiked [ng mL^−1^]	Found [ng mL^−1^]	SD [ng mL^−1^]	SR [%]	Recovery [%]
1	0.20	0.21	0.02	7.5	107
2	1.00	0.99	0.03	3.1	99
3	4.00	4.07	0.14	3.3	102

**Table 3:** Comparison of cortisol concentration in human blood serum and saliva measured by the SPRi method and a standard method.

Medium	Samples Number	Cortisol Concentration [ng mL^−1^]	
		SPRi	Standard Method
Serum	1 2 3 4 5 6 7 8 9	81.0 ± 6.4 103.8 ± 7.7 108.1 ± 5.1 23.9 ± 2.3 45.9 ± 3.2 14.6 ± 2.3 18.6 ± 1.9 110 ± 0.8 78.6 ± 4.2	85.6 101.6 111.6 28.4 48.7 13.9 19.1 105.5 83.4
Saliva	1 2 3 4 5 6 7 8 9	2.6 ± 0.19 7.3 ± 0.54 4.6 ± 0.52 2.1 ± 0.20 3.6 ± 0.36 3.9 ± 0.39 7.6 ± 0.12 1.4 ± 0.07 5.2 ± 0.24	2.8 6.3 5.3 2.7 3.5 4.5 6.9 1.1 6.9

**Figure A2:**
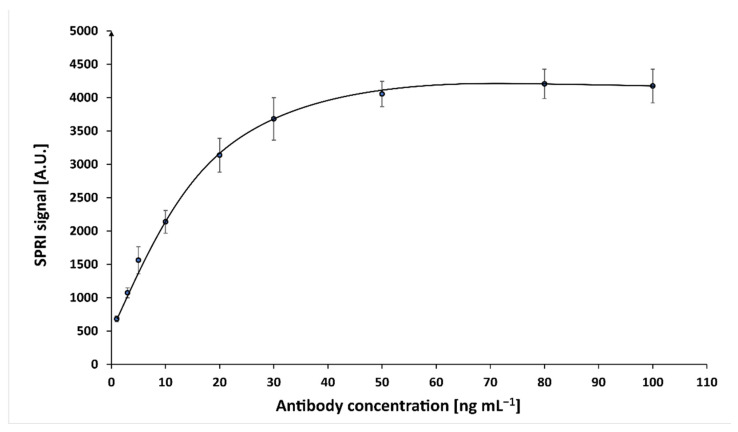
Dependence of the analytical signal on the concentration of mouse monoclonal antibody against cortisol (cortisol concentration 10 ng mL^−1^, pH = 7.4).

## Data Availability

Not applicable.

## Appendix A

### Materials

Cortisol solution (Merck, Darmstadt, Germany), mouse monoclonal antibody against cortisol, leptin (ABCAM, Cambridge, UK), cysteamine hydrochloride, N-ethyl- N′-(3-dimethylaminopropyl) carbodiimide (EDC), progesterone, estradiol, testosterone, cholesterol, human albumin (all SIGMA-ALDRICH, Steinheim, Germany), and N-hydroxysuccinimide (NHS) (ALDRICH, Munich, Germany) were used, as well as absolute ethanol, acetic acid, hydrochloric acid, sodium hydroxide, sodium chloride, sodium carbonate, sodium phosphate, potassium phosphate, sodium acetate, potassium chloride, magnesium chloride (POCh, Gliwice, Poland), HBS-ES solution pH = 7.4 (0.01 M HEPES, 0.15 M sodium chloride, 0.005% Tween 20, 3 mM EDTA), carbonate buffer pH = 8.50–9.86, Phosphate Buffered Saline (PBS) pH = 7.4 (all BIOMED, Lublin, Poland), HBS-ES buffer solution pH = 7.4 (0.01 M HEPES, 0.15 M sodium chloride, 0.005% Tween 20, 3 mM EDTA), photopolymer ELPEMER SD 2054, hydrophobic protective paint SD 2368 UV SG-DG (PETERS, Kempen, Germany) Argon N 5.0 with an Ar content ≥99.999 % was used (Air Liquide Polska Sp. z o.o., Bialystok, Poland).

The bases of the biosensors were chips covered with a layer of gold (Ssens, The Netherlands).

## Appendix B

### Apparatus and Chip Architecture

SPRi Array measurements were performed on a home-made apparatus consisting of a HeNe laser (JDS Uniphase, Edmund Industrial Optics, Barrington, NJ, USA), two glass lenses L1 (f 3 mm) and L2 (f300mm), two polarizers (P1 and P2), a mirror, a prism, and a CCD camera (QICam, QImaging, Surrey, BC, Canada). The apparatus is shown schematically in Figure A1. NIH Image J version 1.42 software was used for evaluation of the SPRi images in 2D form.

The chip is an array of 108 active points. It has nine round measuring sites, each with 12 free gold surfaces. The chip is shown in Figure A1. Using this chip, nine different solutions can be measured simultaneously without mixing the tested samples, and 12 separate SPRi measurements can be performed from a single 3 µL droplet of tested solution.

## Appendix C

### Optimization of Concentration of Mouse Monoclonal Antibody (Receptor)

Optimization of the concentration of mouse monoclonal antibody against cortisol was performed at constant cortisol concentration (10 ng mL^−1^). On the chip, which was covered with cysteamine, activated solutions of antibody in different concentrations were applied. The concentrations of the antibody solutions were within the range 1.00–100 ng mL^−1^. The antibody has been activated with a mixture of EDC (250 nM) and NHS (250 nM) in carbonate buffer (pH8.5). After incubation at 37 °C for 1 h, the antibody was covalently immobilized on the chip, which was then rinsed with water and dried under a stream of argon.

The first SPRi measurement was performed. Next, the immunosensor’s active sites were treated with 10 ng mL^−1^ solution of cortisol for 10 min, rinsed with HBS-ES buffer and water, and dried under a stream of argon, after which the second SPRi measurement was performed. The analytical signal was calculated as the difference of the signal before and after the interaction of the antibody with cortisol. Based on the results, a graph was plotted of the SPRi signal as a function of antibody concentration (Figure A2). A concentration of 50 ng mL^−1^ was selected as the optimal value for further experiments.
